# “My gut feeling is we could do more...” a qualitative study exploring staff and patient perspectives before and after the implementation of an online prostate cancer-specific holistic needs assessment

**DOI:** 10.1186/s12913-019-3941-4

**Published:** 2019-02-12

**Authors:** Amy L. Clarke, Julia Roscoe, Rebecca Appleton, Jeremy Dale, Veronica Nanton

**Affiliations:** Unit of Academic Primary Care, Division of Health Sciences, Warwick Medical School, University of Warwick, Room A115, First Floor, Coventry, CV4 7AL UK

**Keywords:** Qualitative study, Motivations and barriers, Theoretical domains framework, Holistic needs assessment, Information technology, Cancer follow-up, Integrated care

## Abstract

**Background:**

Men surviving prostate cancer report a wide range of unmet needs. Holistic needs assessments (HNA) are designed to capture these, but are traditionally paper-based, generic, and only carried out in secondary care despite national initiatives advocating a “shared care” approach. We developed an online prostate cancer-specific HNA (sHNA) built into existing IT healthcare infrastructure to provide a platform for service integration. Barriers and facilitators to implementation and use of the sHNA were explored from both the patients and healthcare professionals (HCPs) perspectives.

**Methods:**

This qualitative study consisted of two phases. Phase 1 used semi-structured interviews to explore HCPs (*n* = 8) and patients (*n* = 10) perceptions of the sHNA, prior to implementation. Findings were used to develop an implementation strategy. Phase 2 used semi-structured interviews to explore HCPs (*n* = 4) and patients (*n* = 7) experienced barriers and motivators to using the sHNA, 9 to 12 months after implementation. Interviews were audio-recorded, transcribed verbatim and thematically analysed. Themes were mapped to the Theoretical Domains Framework.

**Results:**

HCPs and patients anticipated many benefits from using the sHNA. Barriers to implementation included: confidence to work in depth with prostate cancer patients, organisational and cultural change, and patient factors. Our implementation strategy addressed these barriers by the provision of disease specific training delivered in part by a clinical nurse specialist; and a peer-led IT supporter. Following implementation HCPs and patients perceived the sHNA as beneficial to their practice and care, respectively. However, some patients experienced barriers in using the sHNA related predominately to symptom perception and time since treatment. HCPs suggested minor software refinements.

**Conclusions:**

This work supports the importance of identifying barriers and motivators to implementation, and using targeted action via the development of an implementation strategy to address these. Whilst this process should be on-going, undertaking this work at an early stage will help to optimise the implementation of the sHNA for future trials.

**Electronic supplementary material:**

The online version of this article (10.1186/s12913-019-3941-4) contains supplementary material, which is available to authorized users.

## Background

Prostate cancer is the most common malignancy diagnosed in men living in the United Kingdom [1]. While 10-year survival rates (84%) are encouraging [1], “survivors” live with a broad range of serious consequences because of their disease or the effects of treatment [2]. A survey by Watson et al. [3] indicated that 1 in 3 men experience sexual, urinary or bowel dysfunction at 2 years’ post diagnosis. Men report dissatisfaction with current care, and on-going unmet needs in areas such as intimacy, general information, physical and psychological health [3]. There are currently over 200,000 men living with or beyond a diagnosis of prostate cancer in the UK, and this is expected to increase to over 800,000 by 2040 [4].

Growing numbers of cancer survivors are placing an increasing demand on already stretched specialist services. The National Cancer Survivorship Initiative promotes a shared care approach whereby primary care has greater involvement in the management and follow-up care of prostate cancer [5]. A recent trial conducted in Australia showed that prostate cancer patients prefer a shared care approach, and how integrated models of care can produce similar clinical benefits at a reduced cost compared to specialist care only [6].

A holistic needs assessment (HNA) aims to identify patients’ unmet needs and facilitate the development of person centred care plans [7]. However, current HNAs tend to be paper based, and/or generic and single factor; acting as a potential barrier for shared care and limiting HCPs ability to prioritise concerns [8]. A retrospective analysis of HNAs completed by over 1200 cancer patients indicated that 9 of the top 10 concerns raised were associated with a specific cancer site [7]. This suggests that a generic HNA may not be sufficient to identify the unmet needs of men with prostate cancer.

Digital technology embedded into existing healthcare IT infrastructure has the potential to revolutionise care for cancer survivors. The sharing of an online HNA between generalist and specialist services may facilitate the development of an integrated model of care. However, there is limited evidence available to inform the development of interventions or polices that strive for better care co-ordination in cancer care [9]. Despite the potential benefits of e-health, there appears to be a gap between perceived benefits, versus actual documentation of outcomes and adoption into routine practice [10, 11]. Barriers to the implementation of e-health from the perspective of primary care nurses include: pressure due to increased procedures, risk of de-personalised care, lack of organisational support for training, and questioning of professional expertise [12]. Furthermore, patient barriers to using e-health are commonly reported, with age, illness burden, social support, gender, education and income often cited [13, 14].

We developed an online prostate cancer-specific HNA (sHNA). Built into existing NHS IT infrastructure, the sHNA allows primary and secondary care services the opportunity to review outputs. The sHNA includes a semi-automated care plan designed to help primary care practice nurses coordinate care, as well as targeted resources to enable self-management [15]. Given the known barriers to implementing e-health interventions, and the potential added difficulties of targeting a workforce with traditionally limited experience of prostate cancer and a clinical population of an older age, we felt it necessary to better understand the specific barriers and motivators to the implementation of the online sHNA.

Factors influencing the implementation of e-health into a complex care setting are likely to be multifaceted. We sought a theoretical approach to better understand barriers and motivators to implementation and use of the online sHNA. The Theoretical Domains Framework (TDF) has proved useful in identifying barriers and facilitators to the implementation of many primary care interventions [16–18], and can be applied to both qualitative and quantitative data [19]. It comprises of 14 domains which are illustrated in Table 2.

This paper describes healthcare professionals’ (HCPs) and patients’ perceptions prior to, and after, the implementation and use of the online sHNA within a feasibility study. The aim of the current study was to identify perceived barriers and motivators to implementation and continued use of the sHNA.

## Methods

### Study design

This qualitative study consisted of two phases. Firstly, we explored patients’ and HCPs’ perceived barriers and motivators to the implementation of an online sHNA in primary care (Phase 1). Follow-up interviews were conducted to explore barriers and motivators to implementation and continued use of the sHNA (Phase 2). Data were elicited using semi-structured interviews which were conducted as part of the ICARE-P (Partnership in Prostate Cancer Care) feasibility study [15]. We captured experiences of implementation and use of the sHNA from both generalist and specialist services, as ICARE-P aimed to improve service integration for the assessment and treatment of unmet needs in men with prostate cancer.

This study was approved by the East Midlands - Nottingham 2 Research Ethics Committee (REC reference: 15/EM/0534). Written consent was obtained from all participants.

### Participant characteristics and sampling

**Fig. 1:**
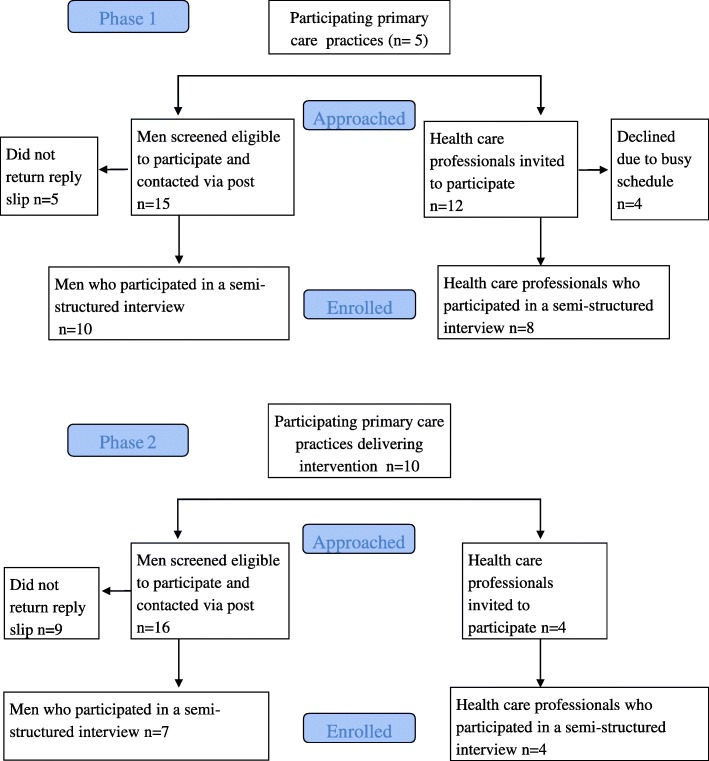
Consort diagram to show participant flow through study for Phases 1 and 2

Participant flow through study is shown in Fig. 1.

### Phase 1: Initial interviews

A convenience sampling method was used to recruit eligible participants from five general practices, participating in ICARE-P. Men were eligible to participate if they had ever received a prostate cancer diagnosis, were able to communicate in English, and were currently under the care of the participating practices. Following screening by the practice, men deemed eligible for inclusion were approached via postal invitations, which included a patient information sheet and an expression of interest reply slip to return via the enclosed pre-paid envelope.

HCPs working within the participating practices were invited to an interview if their role involved the provision of care for men with prostate cancer. To ensure diverse representation we included both general practitioners (GPs) and practise nurses, to gain an overall perspective of practice support for the sHNA.

Identified barriers from Phase 1 were used to inform and refine our implementation strategy for ICARE-P. Strategies utilised proven behaviour change techniques [20] and were decided upon through detailed discussions with the study steering group, including Patient and Public Involvement (PPI) members recruited from local Prostate Cancer Support Associations.

### Phase 2: Follow- up interviews

A purposive sampling method was used to recruit participants who had completed the sHNA at least twice (*n* = 16) over a 9-month period as part the ICARE-P study. Recruitment was undertaken by letter as described above, or email depending on user preference.

All HCPs (*n* = 3 practice nurse) involved in the implementation of the sHNA took part in a follow-up interview. Due to practice recruitment issues with ICARE-P, two of the practice nurses covered multiple intervention sites in their dual role as research nurses. We also invited the clinical nurse specialist (CNS) (*n* = 1), who provided training, on-going advice and accepted referrals from primary care as part of the ICARE-P study, for an interview. GPs perspectives were not explored in Phase 2 due to their limited involvement with the sHNA.

### Data collection

Phase 1: Semi-structured interviews were conducted by VN and RA prior to the implementation of the sHNA. VN is an experienced social scientist with expertise in prostate cancer research, and RA a research associate with a background in psychology. Following these interviews, the sHNA was implemented.

Phase 2: Follow-up interviews were conducted with HCPs and patients who had experience of using the sHNA. Phase 2 interviews were conducted by JR, an experienced researcher with a nursing background, involved in data collection, but not intervention delivery. Phase 2 interviews took place between 9 and 12 months after the sHNA was implemented.

Semi-structured interviews undertaken with patients were conducted face to face in the participant’s home. Interviews with HCPs were undertaken face to face at their place of work or via the telephone. Patient interviews lasted between 12 and 14 min, and interviews with HCPs between 13 to 27 min.

Topic guides were developed in collaboration with PPI representatives. The overarching question themes are summarised in Table 1.

**Table 1:** Topic guide summaries

HCP	Patient
Pre-implementation	
• Experience and level of involvement caring for men with prostate cancer • Views on expanding the role of Primary Care for men with prostate cancer • Experience of using holistic needs assessment in routine practice • Perceived advantages and disadvantages to implementing the sHNA • Anything else	• Experience of prostate cancer • Prior experience of completing a HNA • Experience of IT • Demonstration of resource • Perceived advantages and disadvantages of using the online sHNA • Perceived need for support • Perception of communication between primary and secondary care • Anything else
Post-implementation	
• Experienced advantages and disadvantages of implementing and using the sHNA • Experience of training and support • Perceptions of the software including ease of use • Perceived potential for adoption into routine practice • Anything else	• Experienced advantages and disadvantages of using the online sHNA • Acceptability of the sHNA summary and subsequent nurse consultation • Perceptions of the software including ease of use • Thoughts about future use of sHNA • Anything else

### Data analysis

Interviews were audio-recorded using an encrypted device, and managed on a secure server and professionally transcribed verbatim. Transcripts omitted any identifiable information to ensure anonymity.

To ensure rigour, transcripts were independently coded by two researchers, JR and AC, a researcher in health psychology, but no prior study involvement. PPI members (*n* = 2) with knowledge of ICARE-P, either via peer support or steering group involvement, were invited to participate in the initial identification of themes.

Data were analysed using inductive thematic analysis following the steps provided by Braun and Clarke [21]. Identified themes were mapped to the TDF, which was used as a conceptual framework, prior to defining final themes as part of the analysis. This process was facilitated by Nvivo version 11. Themes were not forced to fit one domain. Instead, we developed a thematic map to visualise the relationship between themes, which allowed for further discussion, grouping and re-defining (see Additional file 1 and Additional file 2). Domains deemed useful for organising themes are indicated and described in Table 2.

**Table 2:** Domains of the TDF deemed useful in explaining perceived and experienced barriers and facilitators to implementation and use of the sHNA

	Phase 1		Phase 2	
	HCP	Patient	HCP	Patient
1. Knowledge (Knowledge of condition and rationale of holistic needs assessment)	✓	✓	✓	
2. Skills (Competence and skill development with regards to use of sHNA)			✓	✓
3. Social/professional role and identity (Perceived role of HCPs to work with prostate cancer, from both the HCPs and patient perceptive)	✓		✓	
4. Beliefs about capabilities (Perceived competence to care [HCP only] for men with prostate cancer and use of the sHNA); perceived capabilities to use the sHNA)	✓	✓	✓	✓
5. Optimism (Confidence that the sHNA will be adopted or useful in some way)	✓	✓	✓	✓
6. Beliefs about Consequences (Anticipated or experienced outcomes from implementing the sHNA)	✓	✓	✓	✓
7. Reinforcement (Increasing the probability of a response by arranging a specific dependant relationship)				
8. Intentions (Conscious decision to use the sHNA in a certain way)				
9. Motivation and goals (Outcomes or endpoints that HCPs or patients want to achieve via the sHNA)			✓	
10. Memory, attention and decision processes (Ability to retain thoughts, and make decisions between two or more alternatives)				
11. Environmental context and resources (Circumstances impacting on an individual’s ability to use the sHNA in the intended way)	✓	✓	✓	✓
12. Social influences (Interpersonal processes that cause an individual to change their thoughts or feelings)				
13. Emotion (A complex reaction pattern that may influence the individual’s willingness to use the sHNA)	✓	✓		
14. Behavioural regulation (Anything aimed at managing or changing behaviour e.g. self-monitoring of symptoms using sHNA)		✓		

## Results

Patient and healthcare professional characteristics are indicated in Table 3 and 4, respectively.

**Table 3:** Patient characteristics

Participant Code	Age range (years)	Time since diagnosis (years)	Treatment Type	Number of co-morbidities
Patient 1^a^	66–70	12	Surgery	0
Patient 2^a^	61–65	3	Surgery	2
Patient 3^a, b^	66–70	10	Surgery	1
Patient 4^a^	81–85	15	Radiotherapy	1
Patient 5^a^	81–85	5	Active Surveillance	1
Patient 6^a^	61–65	2	Surgery	0
Patient 7^a^	76–80	3	Radiotherapy & ADT	1
Patient 8^a^	76–80	5	ADT	1
Patient 9^a^	76–80	10	Surgery & ADT	0
Patient 10^a^	71–75	2	ADT	1
Patient 11^b^	76–80	13	Radiotherapy	1
Patient 12^b^	76–80	4	Radiotherapy & ADT	1
Patient 13^b^	76–80	5	Active Surveillance	0
Patient 14^b^	71–75	13	Surgery	0
Patient 15^b^	71–75	7	Radiotherapy & ADT	1
Patient 16^b^	81–85	2	ADT	2

Abbreviations: *ADT* Androgen Deprivation Therapy

^a^Denotes patients interviewed in Phase 1

^b^Denotes patients interviewed in Phase 2

**Table 4:** Healthcare professional characteristics

Healthcare professionals	Phase 1	Phase 2
GP	4	0
^a^Practice Nurse	4	3
CNS	0	1

Abbreviations: *GP* General practitioner, *CNS* Clinical nurse specialist

^a^Practice Nurse HCP 3 participated in Phase 1 and Phase 2 interviews

### Phase 1: Pre-implementation facilitators

#### Positive appraisals and anticipated benefits (Perceived consequences and optimism)

Prior to implementation of the sHNA both HCPs and patients anticipated a number of benefits.

HCPs anticipated that the sHNA would help to facilitate patient involvement in their care, resulting in patients being better able to express their concerns. The sHNA was perceived as a potentially useful conversation starter, and a way of focusing consultations.

*“I think it's a really good idea …it gives the patient a little bit of not power as such but good involvement, so it's, you know, very much patient centred care and we can … spend our time really on the bigger issues…” (HCP3, Nurse).*

HCPs were keen to trial the sHNA as a way of enabling shared care, feeling that the sHNA would allow them greater insight into the patient’s specialist care.

*“I think it's good. I don’t think we have a lot of contact with prostate services in hospitals…so that's a shame because …patients will come and you will have to wait for them to tell you what happened at their appointment” (HCP3, Nurse).*

Similarly, despite describing no prior experience with using an HNA, patients felt that the resource would be helpful in prioritising their concerns. Perceiving this as a time saver for HCPs and a way of providing them with a better picture of their overall health. Whilst some patients felt well informed about their condition, others described information needs and a desire for practical advice which they perceived could be gained by using the sHNA. After viewing the resource some participants felt optimistic that it may benefit even those with limited IT skills.

*“…a practical advantage is in the sense that I went to the nurse you know, with perhaps more specific questions, saving her time in a way, as well as helping me with what she was going to say” (Patient 9). *

*“…it would be like somebody sitting next to you and being able to ask them the questions, and they could answer it to you, which at the moment I’m just getting nothing at all really…when I do get a question, I just sort of shelve it because there’s nobody really I can ask” (Patient 10).*

*“It’s very good. I think especially for people who aren’t necessarily that computer literate, oddly enough. Because if you are, you can do your own research, but this actually can guide you” (Patient 6).*

### Readiness for implementation (Optimism, environmental context and resources, behavioural regulation, social/professional role and identity)

#### Alignment with current practice

The sHNA was deemed as potentially beneficial to current practice given the potential for the care plan summary screen to be shared electronically between a patient and their HCPs, and between care providers.

*“I think that would be useful because other healthcare practitioners would like to probably see that [sHNA output] when they consult the patient or have a summary of that” (HCP2).*

For some patients completing the sHNA was aligned with their current self-regulatory practices, such as writing lists to avoid forgetting concerns when consulting with their HCP. Having the opportunity to complete the sHNA at home, prior to their consultation, was optimal for those with IT access.

*“I think it’s a good idea because if you can do it in your own home, you’re more liable to think of things than when you go to the doctors. The reason why I have a list is the doctor tells you about the one thing, you’ve then forgotten the other things that …you wanted to ask…” (Patient 3).*

#### Perceived sense of responsibility

HCPs acknowledged a recent increase in their level of involvement with prostate cancer. However, they felt that more could be done in primary care for men with prostate cancer. Nurses perceived themselves as best placed to provide holistic follow-up care for prostate cancer patients and felt they “could offer a really good service” if able to decide upon consultation length themselves (HCP3, Nurse).

*“I think it is crucial that patients are taken out of the hospital system so that we can look after them in primary care…” (HCP2, GP).*

*“I think we're best placed to do it. We do chronic care for most things don't we; why can't we stick prostate on the end” (HCP6, GP).*

*“…I’d probably increase my workload for prostate by a third…so it could be absorbed” (HCP5, Nurse).*

### Phase 1: Pre-implementation barriers

### Confidence to work more in depth with prostate cancer patients (Knowledge, perceived capabilities, environmental context and resources)

HCPs described their current lack of specialist knowledge as a potential barrier to implementing the sHNA. Concerns appeared to be amplified by the preconception that cancer patients prefer specialist care. However, most patients felt confident in the care provided by their local practice and favoured the convenience.

*“I mean if you're giving advice and you're asking those questions you've got to know how to respond to them haven't you…” (HCP6, GP).*

*“…we probably don't do enough, but not sort of fault on anybody's part, I think it's possibly patient's choice or all sorts of reasons why people prefer to go to secondary care...” (HCP1, Nurse).*

*“…the nurse that gave me the injection, she did say she was on this programme. And I did feel that she was in the loop, you know what I mean?” (Patient 10).*

Furthermore, HCPs felt their level of involvement was reliant on the guidance they received from specialist services.

*“…we still need the consultant to give us guidance…so we do need joined up care” (HCP2, GP).*

### Organisational and cultural change (Environmental context and resources, emotion, knowledge)

#### Building capacity

Nurses described a desire to become more knowledgeable in prostate cancer follow-up and emphasised the importance of undertaking training. However, funding and current lack of relevant training were perceived as barriers to implementation.

*“…prostate cancer isn’t just another area of patients that I see…it would be nice if occasionally [we] could have an update …” (HCP3, Nurse).*

*“… to do these sorts of things well you've got to train up nurses, you've got to train up staff, you've got to train up reception staff, you've got [to] chase patients, it doesn't come free of charge” (HCP6, GP).*

#### Previous experience of using a HNA

Few HCPs described using a HNA as part of their routine practice. One GP perceived patient reported outcome measures negatively, fearing loss of face to face contact. The same fear was raised by some patients.

*“No we don't value them one iota, no. I find them a barrier because asking someone to go away and fill in a piece of paper and come back in just doesn't do it for me…” (HCP6, GP).*

*“…I just feel that sometimes, you know, if you can talk on a one to one with somebody, that is the big thing to me” (Patient 1).*

#### Limited involvement with secondary care

HCPs felt that current communication between primary and secondary care was often inadequate, and nurses felt they lacked information about who to contact for support in secondary care. For some patients this resulted in a perceived lack of care continuity.

*“I don’t think there's a clear quick pathway as such. I'm sure there are urology CNS's out there who we can contact; I'm not sure… it's not usually on the letter as to who I need to speak to” (HCP2, GP).*

*“I mean…the only link between the hospital and the GP surgery is the injection, you know” (Patient 10).*

### Patient factors (Beliefs about capabilities and environment context and resources)

HCPs and patients perceived similar barriers to patient engagement with the sHNA including: age, language difficulties, visual impairment, concentration difficulties due to pain, IT skills and access, and patient capability or willingness to make decisions about their own health. One patient feared that the sHNA may act as a reminder of their condition.

*“...with multi, you know, so many different languages going on in our city I think we're kidding ourselves if we think everybody can understand our lovely questionnaires…” (HCP6, GP).*

*“Well it would probably benefit other people. Not myself, but you know, I’m a bit reluctant to use the computer” (Patient 4).*

*“…to be honest with you, I hope I don’t have to reach that sort of stage …I don’t know what the future offers” (Patient 7).*

HCPs were particularly sceptical about the suitability of a digital platform; however, patients with limited IT skills felt that this barrier could be overcome with *“a bit of guidance”* (Patient 1).

### Development of strategic implementation strategy

Using the TDF we identified three key barriers to implementation: 1) lack of disease specific knowledge (knowledge and perceived capabilities); 2) limited involvement with secondary care (environment context and resources, and knowledge); and 3) the generational and multilingual gap (beliefs about capabilities).

Implementation strategies were developed based on proven behaviour change techniques [20]. To address barriers 1 and 2 we provided practice nurses with the opportunity to undertake prostate specific training, which was delivered in part by a (CNS) (provision of information from a credible source and social support) at the secondary care host site. Training included workshops and observation opportunities at the multi-disciplinary team meeting and clinic. Content covered information about prostate cancer diagnosis, treatment pathways, symptoms and side effects. This was designed to help practice nurses gain confidence in their clinical decision making and build effective relationships with secondary care.

Barrier 3 was addressed by the provision of an IT peer-supporter (known in the study as an ITmate) who would be made available to men expressing concerns regarding IT access or skills. The ITmate made home or local practice visits and provided men with a tablet device (restructuring environment), and support to use the sHNA (demonstration of the behaviour). Unfortunately, it was not deemed feasible to address multilingual barriers at this stage in the development of the online sHNA.

### Phase 2: Post implementation facilitators

### Perceived value and impact on care (Beliefs about consequences, motivation, optimism)

Following the implementation of the sHNA, both HCPs and patients described benefits from using it. HCPs appreciated the forewarning about men’s needs which they received as an electronic output from the sHNA, which was available online following patient completion of the assessment. This allowed them to feel more prepared and better able to provide a focused consultation.

*“…it picks up those red flags so that it, it just cuts out time, you’re discussing then with the patient what the patient wants to discuss…” (HCP9, Nurse).*

Men valued the more involved approach to their care and felt the sHNA had encouraged them to reflect more deeply on their needs.

*“…it’s a brilliant way of them being able to say to people what is wrong with you. Otherwise if you sit at home and …as I say when you’re doing nothing your minds wandering…it’s a good idea, it’s excellent” (Patient 16).*

*“…it [sHNA] did make …me consider my situation a little bit more in terms of detailed things …that weren’t necessarily specifically to do with the condition itself…” (Patient 15).*

The sHNA facilitated an opportunity to raise unmet needs that were beyond routine clinical questioning e.g. familial prostate cancer concerns. For men, these reinforced perceptions of good care and helped them to feel comforted.

*“…we can say to them is there anything you want to discuss and then they ring you about these things that you would never have asked them…” (HCP3, Nurse).*

*“…it was a reinforcement of the things that I already had available to me and it was a comfort to know …that I’d got reliable medical people …available to help me if I needed it…” (Patient 15).*

HCPs described feeling connected to more patients, and improved relations with specialist services. Practice nurses felt supported by having direct line access to the CNS which simplified processes such as referrals. The CNS also valued the enhanced communication, feeling it beneficial for reducing adverse events.

*“…I’ve got his [CNS]…direct number which like I say I would have got there eventually but there’s a lot of toing and froing and when you’re in practice it’s having the time…” (HCP3, Nurse).*

*“…yeah it’s just an extra… strategy to you know keep… men more on an even keel…” (HCP11, CNS).*

HCPs felt that the sHNA added value to routine care and could be expanded to other cancer sites. Similarly, patients felt the sHNA might benefit future cancer survivors.

*“… it would be lovely if it all carried on and you could continue using it in lots of different, you know in other areas as well...” (HCP3, Nurse).*

*“…it’s nice and err… I think some… people are going to get benefit…good things are going to come out of it…” (Patient 12).*

### Experienced shift in boundaries of professional role and enhanced understanding (Knowledge, skills and social/professional role and identity)

#### Expansion of boundaries and increased understanding

Using the sHNA reinforced practice nurses’ understanding of the importance of their role in alleviating some of the pressures on specialist services. Following the training and observation opportunities, nurses described greater confidence in their capabilities, and knowing when to refer patients for specialist care.

*“…there is a need for something here now because they’re… 10, 15, 20 years’ post diagnosis…[and] don’t need that immediate bit from the secondary care intervening, it can be looked after in primary care but just know that secondary care is there if you need to refer them...” (HCP9, Nurse).*

#### Practise and support

Confidence to navigate and use the sHNA grew with practise for both HCPs and patients. Overall the resource was described as straightforward to use, although HCPs valued on-going IT support. The ITmate was considered essential for continued use by those who had required this service.

*“I mean it took a bit of getting use to …but now I know exactly where I need to go to get the information I need...” (HCP3, Nurse).*

*Interviewer: “I mean is it something [sHNA] you’d consider doing in the future…?”*

*Participant: “Well if you provided [ITmate]” (Patient 13).*

### Phase 2: Post implementation barriers

### Experience of software (Environmental context and resources, beliefs about capabilities)

#### Connectivity

Connectivity speed and issues logging onto the software were considered a barrier to continued use at one practice. This was a bandwidth problem that related to practice infrastructure.

*“…if the GPs or the nurses could just open it up, click on it, fine…but at the moment the way it’s … taking time… [I get] frustrated and well I’m not bothering using that because it’s not quick enough…” (HCP9, Nurse).*

#### Content and functional issues for refinement

HCPs suggested refinements to improve acceptability. These included a copy and paste function to transfer information easily into GP electronic patient records, access to previous care plans, reminders that consultations were due, and adapting the “red flag function” to better identify new versus historic needs. HCPs also described how other electronic systems they are familiar with return to the homepage after clicking save, the differing save function was described as slightly confusing at times.

*“…the way the questions are worded it almost needs to say…have you had any new back pain or any new symptoms because a lot of them will have already have been addressed years ago” (HCP9, Nurse).*

*“Yeah, it just needs to, once you’ve clicked on save changes it needs to take you back to the first screen” (HCP9, Nurse)*

Patients described some difficulties with the time warnings on each section, indicating these as a disincentive for use; and challenges in determining prostate specific symptoms from issues related to older age and other co-morbidities.

*“I suppose with me …there are things going on but I’m getting older and I don’t know whether it’s through old age or…COPD, prostate cancer … rheumatism, you know what I mean?” (Patient 16).*

### Patient factors (Beliefs about capabilities)

#### Symptom perceptions

Patients who felt generally symptom-free or accepting of their symptoms described some struggles completing the sHNA, sometimes perceiving themselves as a “fraud” (Patient 13). Interestingly, some of these participants did describe burdensome symptoms, yet instead of asking for help chose to enlist personal coping mechanisms. One participant felt the sHNA acted as a reminder of their condition, and acknowledged that they preferred to avoid such thoughts, worried about the negative impact on themselves and those close to them.

*“…obviously, you cannot be perfectly OK… I can’t go out and about by myself because of my incontinence …I just keep myself near to toilet and don’t go out …we live with the problems” (Patient 12).*

*“…is not very responsible but if it was going to give the wrong direction, it’s very sad fact, but if it was going in the wrong direction I’m not sure I’d really want to know...” (Patient 15).*

Others felt the tool lacked personal relevance because they were too many *“years down the road”* since diagnosis (Patient 14).

HCPs did not report any patient barriers to using the sHNA.

## Discussion

This study enhances our understanding of perceived barriers and motivators to the implementation and use of an online sHNA from the perspective of both HCPs and men with prostate cancer. Johnston and Karen [22] recently highlighted the need for greater understanding of the factors influencing HNA implementation. Drawing on the TDF we identified domains perceived as influential to this process.

The majority of participants viewed the sHNA positively (optimism) and anticipated a number of benefits from its use (perceived consequences). HCPs recognised their responsibility to care for the growing numbers of cancer survivors (social/professional role and identity) and felt that the sHNA would be a useful conversation starter to elicit unmet needs. Patients felt the sHNA would provide HCPs with a better overview of their health. HCPs described how the software appeared aligned to current practice and valued the idea of being able to share the care plan summary screen with patients and other professionals. Patients felt that completing the sHNA at home prior to consultation would be beneficial; prompting them to consider their needs within a relaxed environment and saving time for HCPs via focused consultations. A HNA is traditionally completed prior to consultation [8], however, usually this occurs in clinic with the potential for support from a HCP. These findings demonstrate patient acceptability for completing home-based assessments.

Perceived barriers to implementation included a lack of prostate cancer-specific knowledge (knowledge), and limited involvement with secondary care (environmental context and resources). Prostate specific training is not included as part of practice nurse chronic disease management training in the UK, but HCPs described a willingness to further their knowledge and skills. As part of the implementation strategy, practice nurses were given prostate cancer-specific training, delivered in part by a CNS. Training was highly valued and targeted key areas required for improved care integration e.g. shared clinical priorities [23]. Following implementation, HCPs described greater confidence in their ability to identify unmet needs and coordinate the care of men with prostate cancer. Our work supports the feasibility of delivering prostate specific training to practice nurses [24]; and highlights training to be an essential component for implementing an online sHNA in primary care. To be most beneficial HCPs recommended that training be delivered in close temporal proximity to implementation, and be supported with refresher sessions.

Prior to implementation patient factors including age, language, psychological avoidance and desired level of autonomy (perceived capabilities); and access to IT devices (environmental context and resources); were perceived as barriers to patient engagement by HCPs and patients. Higher attrition rates in online intervention arms of trials may partly be due to participants experiencing problems with the digital nature of the trial [25]. After implementation as part of the wider study, men with reduced access or lower IT skills were offered individual support from the ITmate, with 3 of 7 men interviewed for Phase 2 having requested the additional support. This model of support was valued by patients and deemed essential for their continued engagement. Whilst most men described the sHNA as straightforward to use, the ITmate may offer an acceptable model to improve patient engagement and retention with digital interventions.

Information seeking and avoidance (emotion) were not addressed prior to implementation and were perceived by one patient as a barrier to future use in Phase 2. Avoidance of information has previously been associated with the need to maintain hope, whereby individuals enlist avoidance as a coping mechanism to reduce the risk of uncovering information perceived threatening [26]. Patients wanting to avoid further health information, also demonstrated a preference for a paternalistic model of care. Digital health is transforming healthcare, and shared decision making will replace the paternalistic model [27]. Therefore, helping patients to view information as empowering and necessary to make informed decisions about their health is vital.

Symptoms perception (belief about capabilities) was experienced as a barrier to continued use by some men. Men experiencing few symptoms perceived the sHNA to lack relevance in the context of their cancer experience, however, acknowledged that the resource may have been beneficial if implemented closer to treatment. This finding is supported by a recent synthesis of current research detailing the feasibility of web-based interventions in patients with cancer, which indicated that interventions offered sooner may be of more benefit to patients [25]. Others appeared stoic when discussing physical symptoms, seemingly having made a trade-off, accepting the sacrifice of certain functional abilities for survival. It is widely known that men with prostate cancer are reluctant to discuss health concerns [28] however, the mechanisms underpinning this are not fully known. Suppression and minimisation of needs is a common coping strategy for managing threatening situations among men [29] but is likely to produce undesirable outcomes with restricted emotion being linked to poorer psychological adjustment [30]. Therefore, whilst most men felt the online sHNA allowed them to consider and disclose their unmet needs, a greater understanding of symptom perception in prostate cancer may help to identify certain men who may require additional support.

Following implementation, HCPs and patients described a number of benefits (perceived consequences) from using the sHNA. HCPs described feeling more prepared prior to consultations, and patients felt better able to explore their holistic needs and comforted by the extra focus. Barriers to use reflected contextual issues such as frustrations with the IT software (environmental context and resources), with one practice nurse describing connectivity speed and system access as a barrier. Moreover, HCPs recommended refinements after using the software to enhance its acceptability in practice, these included access to previous care plan summaries, and a copy and a paste function to electronic medical notes. Whilst refinements were indicated to improve the online resource, HCPs in the current study reported few workflow barriers related to using the sHNA in their practice. In contrast, Henry [31] described challenges when encouraging clinical nurse specialists to use a structured consultation format during the development and testing of an online HNA. However, the author reported that new members of staff valued a more structured approach to assessment and consultation [31] . Limited previous experience with HNA among practice nurses involved in the current study, may be a reason for fewer perceived barriers. Nurses may have been less able to identify specific barriers or motivators related to technology, with not having experienced the use of a generic paper-based HNA in their practice. As such some of the findings in this study relate more to the general implementation of HNA in primary care.

Patients recommended additional questions designed to help identify prostate related symptoms from other co-morbidities, and the ageing process. HCPs also described some difficulty in determining prostate cancer-specific symptoms, and felt that red flags should be limited to new concerns not currently under investigation. This feedback highlights the potential difficulties of combining a prostate cancer-specific and holistic needs assessment. However, given the high level of multi-morbidity among cancer patients and survivors [32], further investigation is warranted.

### Limitations

This is the first study to explore issues influencing implementation and use of an online sHNA in primary care. Interviews were not designed to be longitudinal, and as such we were unable to track individual perspectives from Phases 1 to 2, given that not all participants participated in both. Instead, we aimed to explore broad collectivist perspectives, as indicated by our sample population in Phase 1; where we collected views from GPs as well as practice nurses. Practice nurses with a dual research role had no existing relationship with patients at some intervention sites, thus potentially influencing their views around the use of sHNA as part of routine practice at these sites. Furthermore, low participation (33%) in Phase 2 interviews may have indicated a self-selecting group of patients with fewer barriers to using the sHNA. As aforementioned attrition rates are often higher in online interventions among patients who experience problems with the digital nature of trials [25] . Whilst retention to the feasibility trial is beyond the scope of this paper, it is important to consider that the barriers reported may not be fully representative of all trial participants. Although, 3 of 7 men did request additional support suggesting that differing IT skills were represented. Finally, we explored perspectives around implementation and use of a sHNA as part of a feasibility trial. However, early investigation of factors influencing implementation is important, and provides insight for optimising implementation for evaluation as part of a future trial.

## Conclusion

This qualitative study highlights key individual and organisational factors important for the implementation and use of an online sHNA in primary care. The most salient barriers to implementation were HCPs lack of specific prostate cancer training, limited involvement with secondary care and patient factors related to perceived capabilities and access to IT devices. The provision of additional training and the use of an ITmate model may help to overcome these barriers, enhancing implementation and use. We have shown that an online sHNA delivered in primary care is acceptable and perceived to be beneficial by patients and HCPs. However, minor refinements were indicated and patients may benefit most from using the sHNA if implemented at diagnosis. These findings will inform our implementation strategy for future trials and help to enhance the adoption of the sHNA into routine practice, should its effectiveness for identifying and addressing the unmet needs of men with prostate cancer be demonstrated.

## Additional files

Additional file 1: Thematic map for Phase 1 interviews. Thematic map to visualise the relationship between themes in Phase 1. (PDF 29 kb)

Additional file 2: Thematic map for Phase 2 interviews. Thematic map to visualise the relationship between themes in Phase 2. (PDF 17 kb)

## Abbreviations

CNS: Clinical nurse specialist
GP: General practitioner
HCPs: Healthcare professionals
HNA: Holistic needs assessment
ICARE-P: Partnership in prostate cancer care
PPI: Patient and public involvement
REC: Research Ethics Committee
sHNA: Online prostate cancer-specific holistic needs assessment
TDF: Theoretical domains framework
