# Supplementary figures and images for: “My gut feeling is we could do more...” a qualitative study exploring staff and patient perspectives before and after the implementation of an online prostate cancer-specific holistic needs assessment
Source: BMC Health Serv Res. 2019 Feb 12;19:115. doi: 10.1186/s12913-019-3941-4 (PMC6373080; doi:10.1186/s12913-019-3941-4)

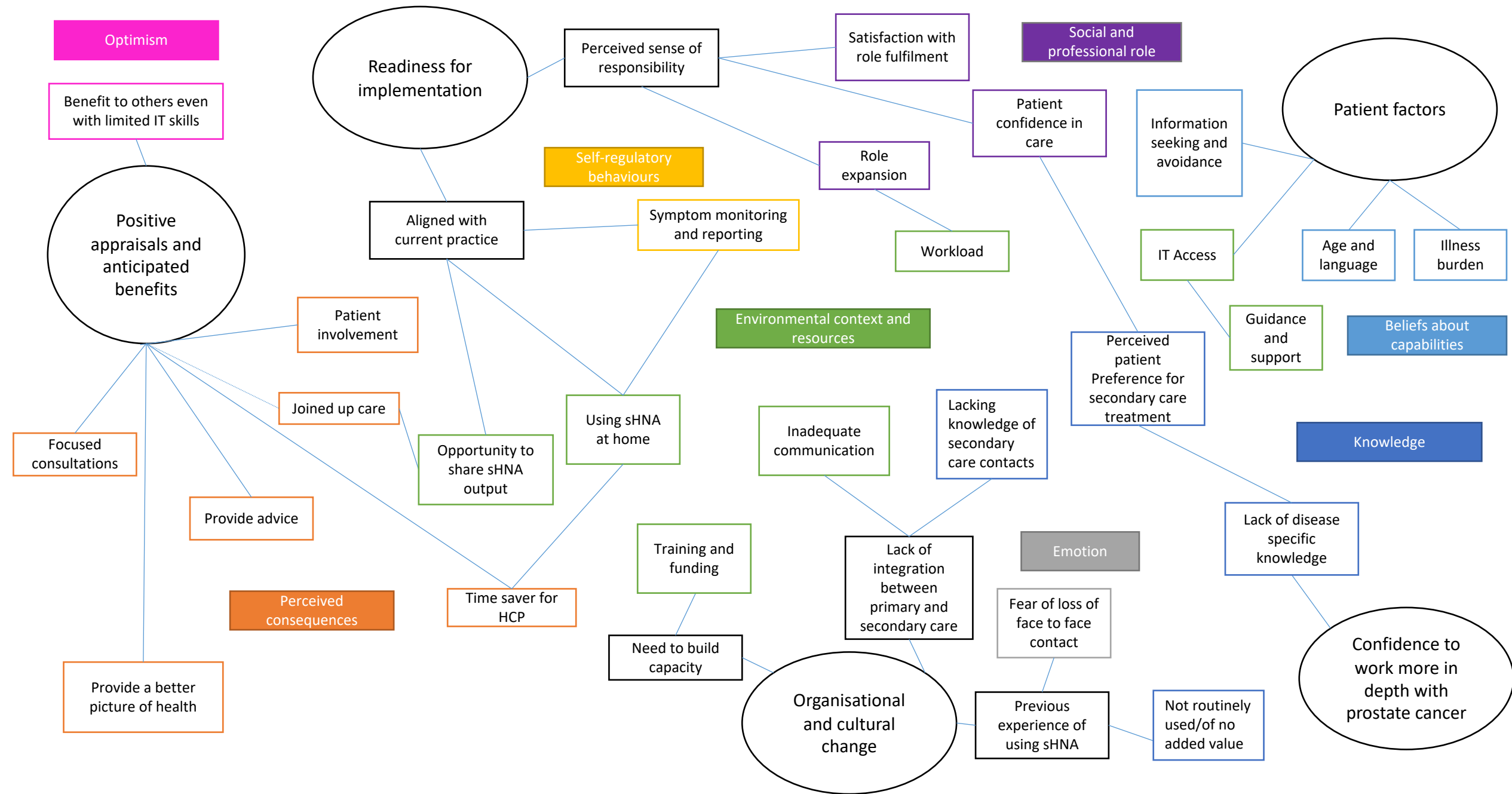

Supplement: Supplementary file 1 — Thematic map for Phase 1 interviews. Thematic map to visualise the relationship between themes in Phase 1. (PDF 29 kb) [file 12913_2019_3941_MOESM1_ESM.pdf]

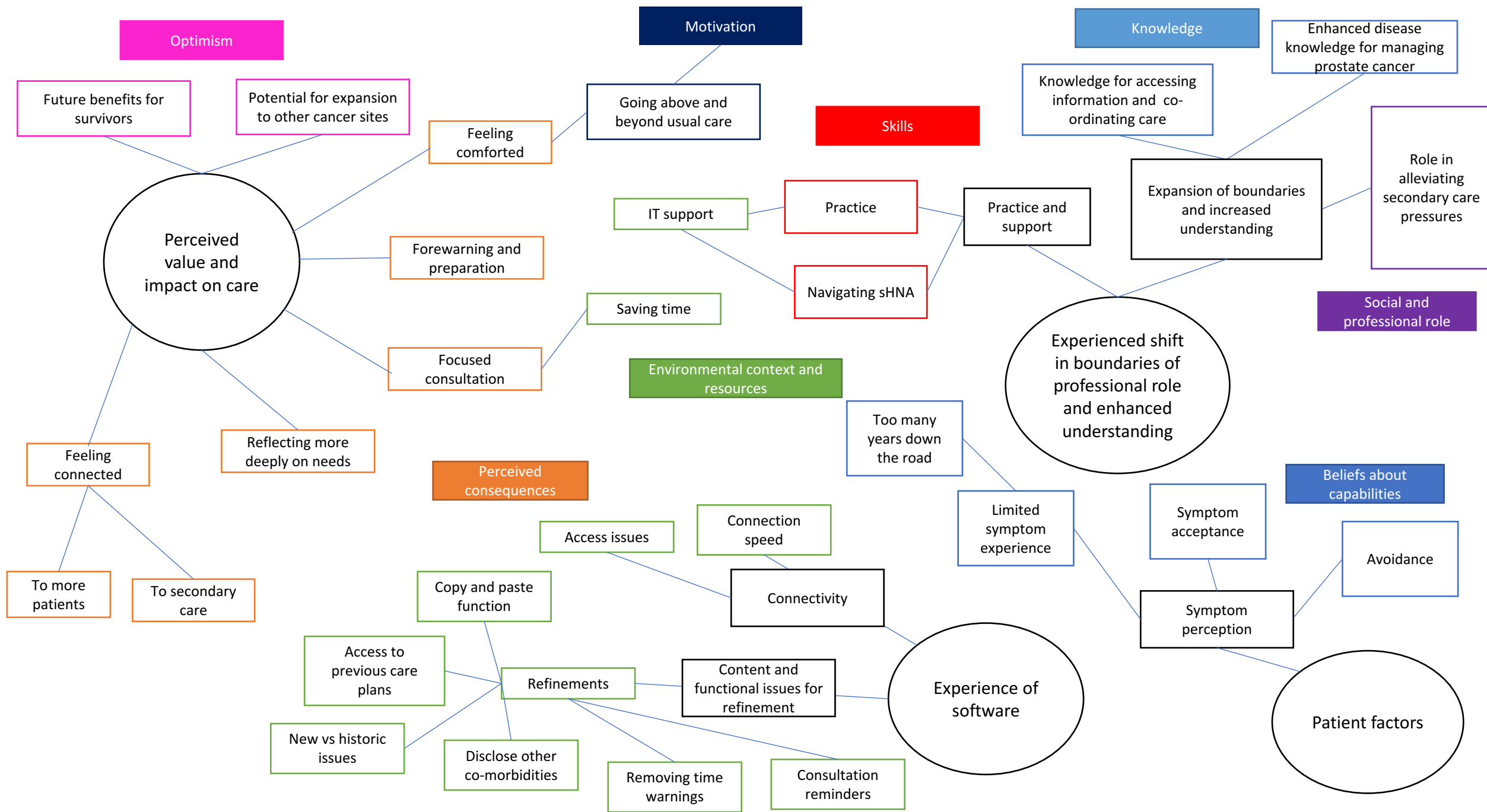

Supplement: Supplementary file 2 — Thematic map for Phase 2 interviews. Thematic map to visualise the relationship between themes in Phase 2. (PDF 17 kb) [file 12913_2019_3941_MOESM2_ESM.pdf]
